# Indigenous and commercial isolates of arbuscular mycorrhizal fungi display differential effects in *Pyrus betulaefolia* roots and elicit divergent transcriptomic and metabolomic responses

**DOI:** 10.3389/fpls.2022.1040134

**Published:** 2023-01-09

**Authors:** Yadong Shao, Shangtao Jiang, Haiying Peng, Han Li, Peigen Li, Rou Jiang, Wenyi Fang, Tingsu Chen, Gaofei Jiang, Tianjie Yang, Savithri U. Nambeesan, Yangchun Xu, Caixia Dong

**Affiliations:** ^1^Jiangsu Provincial Key Lab of Solid Organic Waste Utilization, Jiangsu Collaborative Innovation Center of Solid Organic Wastes, Educational Ministry Engineering Center of Resource-Saving Fertilizers, Nanjing Agricultural University, Nanjing, Jiangsu, China; ^2^Microbiology Research Institute, Guangxi Academy of Agricultural Sciences, Nanning, Guangxi, China; ^3^Department of Horticulture, 1111 Miller Plant Sciences, University of Georgia, Athens, GA, United States

**Keywords:** pear plant, arbuscular mycorrhizal fungi, plant growth, mineral nutrient accumulation, metabolomic, gene responses

## Abstract

**Background:**

Arbuscular mycorrhizal fungi (AMF) are beneficial soil fungi which can effectively help plants with acquisition of mineral nutrients and water and promote their growth and development. The effects of indigenous and commercial isolates of arbuscular mycorrhizal fungi on pear (*Pyrus betulaefolia*) trees, however, remains unclear.

**Methods:**

*Trifolium repens* was used to propagate indigenous AMF to simulate spore propagation in natural soils in three ways: 1. the collected soil was mixed with fine roots (R), 2. fine roots were removed from the collected soil (S), and 3. the collected soil was sterilized with 50 kGy ^60^Co γ-radiation (CK). To study the effects of indigenous AMF on root growth and metabolism of pear trees, CK (sterilized soil from CK in *T. repens* mixed with sterilized standard soil), indigenous AMF (R, soil from R in *T. repens* mixed with sterilized standard soil; S, soil from S in *T. repens* mixed with sterilized standard soil), and two commercial AMF isolates (*Rhizophagus intraradices(Ri)* and *Funneliformis mosseae* (*Fm*)) inoculated in the media with pear roots. Effects on plant growth, root morphology, mineral nutrient accumulation, metabolite composition and abundance, and gene expression were analyzed.

**Results:**

AMF treatment significantly increased growth performance, and altered root morphology and mineral nutrient accumulation in this study, with the S treatment displaying overall better performance. In addition, indigenous AMF and commercial AMF isolates displayed common and divergent responses on metabolite and gene expression in pear roots. Compared with CK, most types of flavones, isoflavones, and carbohydrates decreased in the AMF treatment, whereas most types of fatty acids, amino acids, glycerolipids, and glycerophospholipids increased in response to the AMF treatments. Further, the relative abundance of amino acids, flavonoids and carbohydrates displayed different trends between indigenous and commercial AMF isolates. The *Fm* and S treatments altered gene expression in relation to root metabolism resulting in enriched fructose and mannose metabolism (ko00051), fatty acid biosynthesis (ko00061) and flavonoid biosynthesis (ko00941).

**Conclusions:**

This study demonstrates that indigenous AMF and commercial AMF isolates elicited different effects in pear plants through divergent responses from gene transcription to metabolite accumulation.

## Background

Pear(*Pyrus* sp.) is one of the most economically important fruit crops throughout the world. In pear cultivation, agricultural intensification, especially by increased use of fertilizers and pesticides has led to increased fruit yield (Foley et al., 2011). However, these practice reduce the abundance of keystone taxa and their network complexity in the root microbiome (Banerjee et al., 2019), which in-turn decreases microbial communities and impacts the soil ecological balance. Microbial communities play an indispensable role in nutrient cycling, organic matter decomposition, soil aggregate stabilization, and resistance to biotic and abiotic stress, demonstrating their importance in maintaining the multifunctionality of terrestrial ecosystems (Delgado-Baquerizo et al., 2016). AMF have been used as an alternative to chemical fertilizers to mitigate the impact of agricultural intensification on the soil microbial community, to restore soil ecological balance, and to improve plant growth and yield.

AMF form beneficial obligate symbiotic relationships with majority of plants, largely due to their prevalence in terrestrial ecosystems. In general, AMF hyphae exclusively colonize the root cortex and form an arbuscule, a highly branched structure, inside cortex cells (Berruti et al., 2013). AMF use their fine and extended extraradical hyphal network to explore parts of the soil volume that are not accessible by plant roots. This exploration by AMF allows for mineral and water uptake into plants in exchange for carbohydrates and lipids (Van't Padje et al., 2021), and release of photosynthates into the root zone by plants (Kaiser et al., 2015). Further, AMF can improve plant growth, root morphology, regulate host gene expression and cellular metabolism through various signaling molecules (Lopez-Raez et al., 2010; Rivero et al., 2015; Hill et al., 2018). Previous studies have shown that AMF can regulate the expression of genes associated with root development to promote plant root development, promote the expression of root transporter genes, and improve acquisition of mineral elements by plants (Liu et al., 2022; Wu et al., 2022). In a complimentary way, plant roots can regulate the colonization of AMF by changing the synthesis of its metabolites including that of fatty acids, flavonoids and signaling molecules (Larose et al., 2002; Jin et al., 2016; Kameoka et al., 2019).

There are limited studies on inoculations by commercial AMF isolates and their efficacy in orchard systems. Generally, studies indicate that commercial AMF isolates displayed growth promotion effects on a variety of plants. However, some studies also show that commercial AMF isolates inoculated into orchards can be inhibited by some strains of local bacterial communities, reducing their beneficial effects in the rhizosphere (Cruz-Paredes et al., 2020). Also, compared with multiple AMF, single strain inoculations are more likely affected by local microorganisms, limiting their colonization (Compant et al., 2010). The efficacy of mixed indigenous AMF composed of multiple AMF was more stable than that of single AMF isolates. A previous study showed that the largest and second strongest determinants of AMF community composition were the pool of available AMF and the functional traits of plant hosts, respectively (Smilauer et al., 2020). In long-term symbionts, host plants and AMF select one another to achieve optimal exchange strategy of carbohydrates and nutrients (Noe and Kiers, 2018). Different AMF taxa provide functional complementarity and differential nutritional benefits to the host plants. Thus it is expected that high community diversity is more beneficial for host plants than individual AMF isolates (Wagg et al., 2011; Huang et al., 2019). Application of indigenous AMF in pear orchards can not only promote the growth of pear seedlings, but also maintain the ecological balance of pear orchard soils, reduce the application of chemical fertilizer in agriculture, and maintain the diversity of soil microbial function. However, there are only a few studies on the effects of indigenous AMF on the AMF community composition in pear orchards (Yoshimura et al., 2013; Huang et al., 2019). And the effects of indigenous AMF on pear trees have hardly been studied. Previous study used *Trifolium repens* (It is often used to propagate AMF) as propagation material to propagate AMF spores in rhizosphere soil and root segments of citrus orchard in different ways and found that *T. repens* was an effective way to propagate indigenous AMF spores (Wu et al., 2019). In this study, *T. repens* was used as plant material to propagate AMF in rhizosphere soil and root of pear orchard to study the effects of indigenous AMF on pear trees.

*Pyrus betulaefolia* is a common pear rootstock widely used across the world and displays several disease resistance and nutrient efficiency characteristics (Yang et al., 2020). In this study, we sought to determine the effects of various AMF applications on *P. betulaefolia* plant growth, root morphology, mineral nutrient accumulation, metabolites and transcriptome responses. We hypothesized that: (1) the indigenous microorganisms or plant-specific metabolites in the orchard pear tree root affect the propagation and function of indigenous AMF; (2) the combined effects of indigenous AMF are more beneficial than commercial AMF isolates in enhancing pear growth; and (3) metabolism and gene expression responses of pear roots are specific to indigenous AMF and commercial AMF isolates.

## Materials and methods

### Soil origin and plant growth

Soil samples mixed with fine roots were collected from the rhizosphere at a depth between 0-20cm at a 15 year-old pear (*P. betulaefolia*) orchard which received combined application of chemical and organic fertilizers without AMF inoculum in Changzhou (31°61′N, 120°05′E), Jiangsu province, China. This orchard had been under active production for three years before collection of soil samples, thus the indigenous AMF community from the orchard soil was not likely disturbed by human factors. The collected soil mixed with fine roots were divided into three parts, the first part containing 20 g fine roots (R), the second part where fine roots were removed manually (S) and the third part (rhizosphere soil without fine roots) sterilized with 50 kGy ^60^Co γ-radiation (CK). The soil was air-dried and sieved (<2 mm) to achieve a high degree of homogeneity and to reduce variability among replicates.

Seeds of white clover (*T. repens*, purchased from farmers markets) were sterilized with 75% ethanol for 10 min and rinsed with distilled water. The white clover seeds (about 0.3 g) were sowed in plastic pots (about 0.25 gallons) containing 300 g soil (R, S and CK) mixed with 700 g sterilized sand and vermiculite (2:1, v:v; 0.11 MPa, 121 °C, 2 h). Each treatment was replicated three times, with a total of nine pots in this experiment. All the pots were grown in a greenhouse set at 70% relative humidity set on a light cycle of 15 h daytime (26 °C) and 9 h nighttime (24 °C) at the College of Resources and Environmental Sciences in Nanjing Agricultural University. Daytime illuminance, supplied by LED lighting was 1350 lx. After sowing, plants were irrigated with 50 mL of deionized water and fertilized every week with 50 mL half of low P (10% standard P concentration) Hoagland solution (pH 6.0). Plants were harvested at 80 d after sowing.

After harvest, the soil of the CK, R and S treatments were retained as mixed inoculum for treatment with *P. betulaefolia*. Seeds of pear plants (collected from wild pear trees, collected in November 2019 at Luoyang (34°32’N, 112°16’E), identified by the corresponding author and preserved in the Jiangsu Provincial Key Lab of Solid Organic Waste Utilization, Nanjing Agricultural University, ID Pb-2019-11) were sterilized with 2.5% of sodium hypochlorite solution for 15 min, rinsed with distilled water, and sown into autoclaved (121°C, 0.11 MPa, 0.5h) gauze for germination at 4 °C. After germination, seedlings were transferred to a growth chamber (16 h light/8 h darkness set at 25 ± 1 °C). After 18 d, pear seedlings with uniform size were selected. Standard soil from Baima National Agricultural Science and Technology Zone of Nanjing Agricultural University, sand and vermiculite were mixed in the volume ratio of 2:2:1, sterilized with gamma radiation (50 kGy ^60^Co γ-radiation). The seedlings were transferred to plastic pots (about 0.6 gallons) containing 3.1 kg standard soil and 300 g of R or S inoculum (main species include *Claroideoglomus* sp. and *Acaulospora* sp., unpublished data). To compare the effects of indigenous inoculum, 3.1 kg of standard soil was mixed with 300 g sterilized CK soil (CK), *Rhizophagus intraradices* (BGC BJ09) inoculum (*Ri*) and *Funneliformis mosseae* (BGC HUN03B) inoculum (*Fm*) (about 90 spores/g, approximately the same as the indigenous AMF spore density) separately. The commercial AM fungal strain *R. intraradices* and *F. mosseae* was provided by the Bank of Glomeromycota in China (BGC). The propagation methods of indigenous AMF and commercial AMF were the same, with the only difference being the original soil from the pear orchard and the commercial AMF substrate. Mycorrhizal inoculums included spores, infected root segments, and propagation substrates. However, after *T. repens* propagation, differences of mycorrhizal inoculums were lower among treatments, which are unlikely to result in differences in soil nutrition. In the actual treatments, the original pear tree soil accounted for a very small percentage (about 3%), further indicating that it had almost no impact on plant nutrition. Pear with all treatments were grown in a greenhouse at Baima National Agricultural Science and Technology Zone of Nanjing Agricultural University. One pear seedling of 18 d was used per pot and each treatment was replicated 4 times, for a total of 20 pots for the five treatments (R, S, CK, *Ri*, *Fm*).

### Plant growth parameters

Plants were harvested after 125 d and roots and shoots were separated. Roots were washed free of soil. Subsequently a portion of roots were frozen in liquid nitrogen and stored at -80 °C for root transcriptome sequencing and analysis. The residual roots were used for root architecture measurements using the LA1600 scanner (Canadian Regent Win RHizo2003b). Next, roots were dried in an oven set at 105°C for 30 min, followed by 75°C for 48 h and the dry biomass was measured. The leaves and stems were separated and the total leaf area was measured using a leaf area meter (SYE-YM02, China). The leaves were dried similar to the root tissue and measured for dry biomass.

### Estimation of AMF colonization and spore density

A portion of residual root samples of *T. repens* and pear were stored in 50% ethanol for determination of mycorrhizal root colonization. Root segments were placed in 10% KOH (w/v) and incubated in a water bath set at 90 °C for approximately 15 min (white clover root) or 1 h (pear root), then soaked in an 10% hydrogen peroxide solution at room temperature, and stained with trypan blue for approximately 10 min. Spores in the rhizosphere soil of *T. repens* were isolated from approximately 30 g of air-dried soil by wet sieving, decanting, and centrifugation (3000 rpm, 2 min) through a 65%, (weight/volume) sucrose cushion. Spores were counted using a stereo microscope (40× magnification, BX43, Olympus, Japan). Soil hyphae were extracted from 5 g air dried rhizosphere soil using the membrane filter technique and stained with typan blue for approximately 10 min (Wu et al., 2019; Jiang et al., 2020). Calculations were performed as below:


Root mycorrhizal colonization(%)=100×(root length infected by AMF/total root length observed)

Arbuscular abundance(%)=100×(the number of infected with the presence of arbuscules/total infected by AMF).

Spore density(#/g)=the number of spores/soil weight

Soil hyphal density(m/g)=the total length of hyphae/soil weight

### Analysis of plant chemical parameters

The leaf, stem and other residual root samples were dried in an oven at 105 °C until constant weight, and then ground into a powder using a tissue lyser (TL2010S, DHS Life Science & Technology, China). The N in these samples was extracted by H_2_SO_4_-H_2_O_2_ method and determined by AutoAnalyzer (AA3, Seal, Germany). The P, K, Ca, Mg and Fe of samples was extracted by mixed acid method (concentrated nitric acid:perchloric acid = 4:1) and measured by the Inductively Coupled Plasma-Optical Emission spectroscopy (ICP-OES, Agilent 710, USA). The mineral accumulation (content) was calculated as the concentration of mineral nutrition × biomass.

### Root metabolites extraction and analysis

After grinding with liquid nitrogen 25 mg of CK, R, S, *Ri* and *Fm* root samples were extracted in 500 μL solution containing methanol:water (3:1). Samples were mixed for 30 s using a vortex, homogenized at 35 hz for 4 min and sonicated for 5 min in an ice-water bath, these steps were repeated three times. Next, samples were incubated for 1 h at -40 °C and centrifuged at 12,000 rpm for 15 min at 4°C. The resulting supernatant was transferred to a fresh glass vial for analysis. The quality control (QC) sample was prepared by mixing an equal aliquot of the supernatants from all of the samples. Each treatment was repeated six times.

LC-MS/MS analyses were performed using an UHPLC system (Vanquish, Thermo Fisher Scientific) with a UPLC BEH Amide column (2.1 mm × 100 mm, 1.7 μm) coupled to Q Exactive HFX mass spectrometer (Orbitrap MS, Thermo). The mobile phase consisted of 25 mM ammonium acetate and 25 mM ammonia hydroxide in water (pH = 9.75) and acetonitrile. The column and auto-sampler temperature were set at 30 °C and 4 °C respectively, and the injection volume was 3 μL. The QE HFX mass spectrometer was used to acquire MS/MS spectra on information-dependent acquisition (IDA) mode in the acquisition software (Xcalibur, Thermo).

The relative abundance of metabolites was calculated using the following formula = 100 × (Peak area of class/Total peak area of metabolites).

### Root transcriptome sequence and analysis

Total RNA of root in S and *Fm* treatments was extracted using commercial kits by Guangdong Magigene Biotechnology Co. Ltd. (Guangzhou, China). RNA quantity was measured using Qubit 2.0 (Thermo Fisher Scientific, MA, USA) and Nanodrop One (Thermo Fisher Scientific, MA, USA). RNA integrity was determined using the Agilent 2100 system (Agilent Technologies, Waldbron, Germany). Whole mRNAseq libraries were generated using NEB Next^®^ Ultra™ Nondirectional RNA Library Prep Kit for Illumina^®^ (New England Biolabs, MA, USA) and sequenced on an Illumina Hiseq Xten platform and 150 bp paired-end reads were generated. Each treatment was repeated four times.

The raw data of fastq format were processed by Trimmomatic (v.0.36) to acquire the clean reads and mapped to NCBI Rfam databases, to remove the rRNA sequences by Bowtie2 (v2.33). Reference genome was *Pyrus × bretschneideri* (Chinese white pear) and downloaded in Genebank. The remaining mRNA sequences were mapped to the reference genome by Hisat2 (2.1.0). HTSeq-count (v0.9.1) was used to obtain the read count and gene function information.

To compare gene expression levels among different genes and different experiments, the RPKM (expected number of Fragments Per Kilobase of transcript sequence per Millions base pairs sequenced) of each gene was calculated. Read count of each gene obtained from HTSeq-count was used for differential expression analysis. Differentially expressed genes (DEGs) of two conditions/groups was performed using the edgeR (v3.16.5, http://www.bioconductor.org/packages/release/bioc/html/edgeR.html) which accounts for the length and the number of genes. The resulting *P*-value was adjusted using Benjamini and Hochberg’s approach for controlling the false discovery rate (FDR). The cut off for differentially expressed genes was set at FPKM ≥ 2, FDR ≤ 0.05 and |log2(fold change)| ≥ 1. KEGG (Kyoto Encyclopedia of Genes and Genomes, http://www.genome.jp/kegg/) enrichment analysis of differentially expressed genes (FDR ≤ 0.05) were implemented by the clusterProfiler (v3.4.4, http://www.bioconductor.org/packages/release/bioc/html/clusterProfiler.html) (Kanehisa, 2019; Kanehisa et al., 2021). KEGG Pathways with FPKM ≥ 10 were selected to study the DEGs of fructose and mannose metabolism (ko00051), fatty acid biosynthesis (ko00061) and flavonoid biosynthesis (ko00941) in this study. The sequence raw data and the relevant data have been uploaded to the figshare database (https://figshare.com/s/b81b2c1bb72c49b2e) and Sequence Read Archive (SRA, BioProject: PRJNA761485).

### Statistical analyses

Statistical analyses were carried out using ‘R’ statistical software (version 4.0.3.), implemented within the RStudio graphical user interface (RStudio Desktop, version 2022.07.0, Posit Software, PBC). The data were analyzed using one-way ANOVA (**Table S2**) to test for significance (*p* < 0.05) and Duncan’s test for multiple comparisons using the R package ‘agricolae’. Illustrations were made using Origin8.5 (Origin Inc., Chicago, USA) and ‘R’ statistical software (version 4.0.3.).

## Results

### Propagation of indigenous AMF in white clover

**Figure 1:**
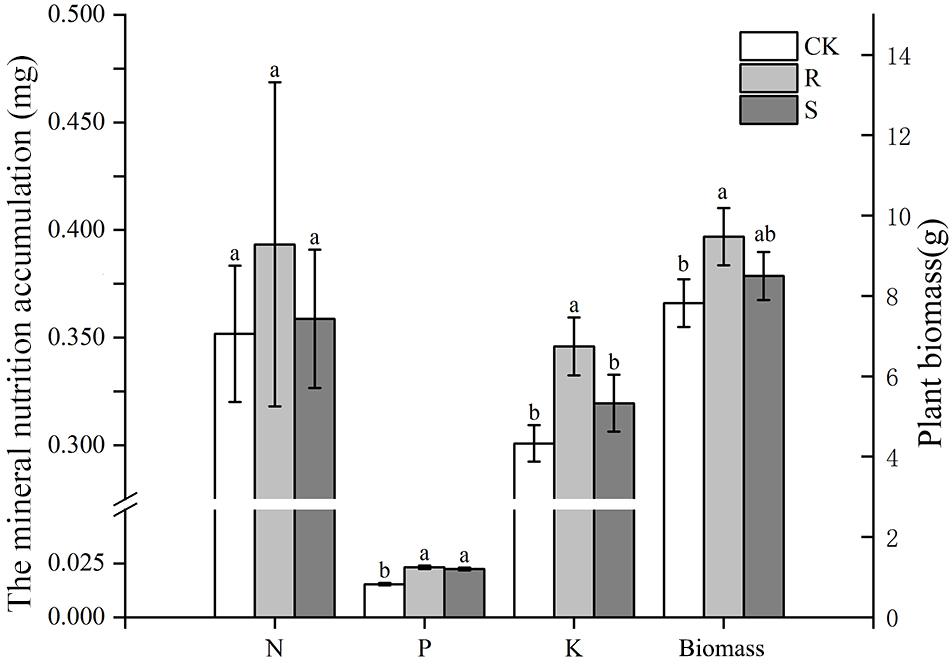
The mineral nutrition accumulation (N, P, and K) and biomass of white clover in different treatment. CK, sterilized soil from rhizosphere mixed with sterilized sand and vermiculite; R, soil and fine roots from rhizosphere mixed with sterilized sand and vermiculite; S, only soil from rhizosphere mixed with sterilized sand and vermiculite. Data (means ± SD, n = 3) followed by different letters indicate that values are significantly different (*p* < 0.05) within the treatments.

Before harvest, root mycorrhizal colonization was observed in the R and S treatments, but not found in CK treatment (**Figure S1**). Root mycorrhizal colonization and arbuscular abundance in S treatment was significantly higher than in the R treatment (**Table 1**). The spore density in the original rhizosphere soil of pear orchard was 21.5 spores/g, which increased significantly after propagation by 300% in R and by 347% in S treatments. The spore density and soil hyphal density after propagation was not significantly different between the R and S treatments (**Table 1**). There were no differences in N and P accumulation in the R and S treatments compared with CK treatment, and K accumulation was higher in the R treatment (**Figure 1**). Further, the dry biomass of the CK treatment was lower than that in the two other treatments (**Figure 1**).

**Table 1:** The mycorrhizal propagation performance between R and S treatment in white clover root and rhizosphere.

Treatment	Root mycorrhizal colonization (%)	Arbuscular abundance (%)	Spore density (#/g)	Soil hyphal density (m/g)
CK	0 ± 0c	0c	0b	0b
R	55.38 ± 4.66b	65.89 ± 4.08b	86 ± 15.28a	0.33 ± 0.07a
S	72.22 ± 10.72a	74.63 ± 2.14a	96 ± 35.47a	0.27 ± 0.05a

CK, sterilized soil from rhizosphere mixed with sterilized sand and vermiculite; R, soil and fine roots from rhizosphere mixed with sterilized sand and vermiculite; S, only soil from rhizosphere mixed with sterilized sand and vermiculite. Data (means ± SD, n = 3) followed by different letters indicate that values are significantly different (*p* < 0.05) within the column.

### Growth performance in pear plants

AMF treatments resulted in increased growth compared with the CK treatment. Shoot dry biomass, plant height, leaf number and leaf area were significantly increased in R, S, *Fm* and *Ri* treatments, compared to CK (**Table 2**). With the exception of shoot dry biomass, which was not significantly different among AMF treatments, the number of leaves, plant height and leaf area were greater in the S treatment.

**Table 2:** The effects of indigenous AMF and commercial AMF isolates on growth performance of *Pyrus betulaefolia*.

Treatment	Shoot dry biomass (g)	Plant height (cm)	Leaves number (#)	Leaf area (mm^2^)
CK	0.05 ± 0.01b	5.50 ± 0.14d	9.00 ± 0.82c	1338 ± 461c
R	0.71 ± 0.32a	18.35 ± 4.72c	26.00 ± 5.48b	9319 ± 3975b
S	0.84 ± 0.27a	29.65 ± 2.70a	33.75 ± 8.26a	14949 ± 2841a
Fm	0.59 ± 0.10a	23.38 ± 2.70b	24.75 ± 2.22b	12953 ± 2437ab
Ri	0.54 ± 0.12a	22.15 ± 0.73bc	25.25 ± 2.06b	11818 ± 2565ab

CK, sterilized soil from CK in *Trifolium repens* mixed with sterilized standard soil; R, soil from R in *Trifolium repens* mixed with sterilized standard soil; S, soil from R in *Trifolium repens* mixed with sterilized standard soil; Fm, *Funneliformis mosseae* inoculum mixed with sterilized standard soil; Ri, *Rhizophagus intraradices* inoculum mixed with sterilized standard soil. Data (means ± SD, n = 4) followed by different letters indicate that values are significantly different (*p* < 0.05) within the column.

### Mycorrhizal colonization and root morphology in pear plants

The CK treatment did not display any AMF colonization in the root of pear plants, while mycorrhizal colonization was significantly affected by the diverse arbuscular mycorrhiza taxa (**Table 3**). Root mycorrhizal colonization among AMF treatments were ranked as S > R > *Fm* = *Ri*. However, the results also revealed that there were no significant differences among all treatments for the diversity of bacterial communities (**Figures S2**, **S3**). All of the root morphology attributes measured were higher in the treatments compared to CK (**Table 3**). Among the various treatments, root dry biomass was higher in *Fm* compared with the R treatment, root length was highest in the S treatment and, root volume and surface area were higher in the indigenous AMF treatments compared to the *Ri* isolate.

**Table 3:** The root mycorrhizal colonization of indigenous AMF and commercial AMF isolates and the effects on root morphology of *Pyrus betulaefolia*.

Treatment	Mycorrhizal colonization (%)	Root dry biomass (g)	Total length (m)	Root volume (cm^3^)	Surface area (cm^2^)
CK	0 ± 0d	0.21 ± 0.05c	2.78 ± 0.22c	0.30 ± 0.04d	31.92 ± 4.11d
R	68 ± 2b	0.45 ± 0.09b	5.76± 1.19b	1.25 ± 0.35a	89.33± 22.09ab
S	80 ± 14a	0.66 ± 0.17ab	7.02 ± 2.57a	1.10 ± 0.24ab	97.62 ± 24.95a
Fm	38 ± 7c	0.81 ± 0.23a	4.40 ± 0.91b	0.79 ± 0.20bc	65.64 ± 12.52bc
Ri	35 ± 7c	0.60 ± 0.08ab	4.70 ± 1.00b	0.66 ± 0.15c	62.28 ± 13.29c

CK, sterilized soil from CK in *Trifolium repens* mixed with sterilized standard soil; R, soil from R in *Trifolium repens* mixed with sterilized standard soil; S, soil from R in *Trifolium repens* mixed with sterilized standard soil; Fm, *Funneliformis mosseae* inoculum mixed with sterilized standard soil; Ri, *Rhizophagus intraradices* inoculum mixed with sterilized standard soil.

Data (means ± SD, n = 4) followed by different letters indicate that values are significantly different (*p* < 0.05) within the column.

### Mineral nutrition accumulation in pear plants

**Figure 2:**
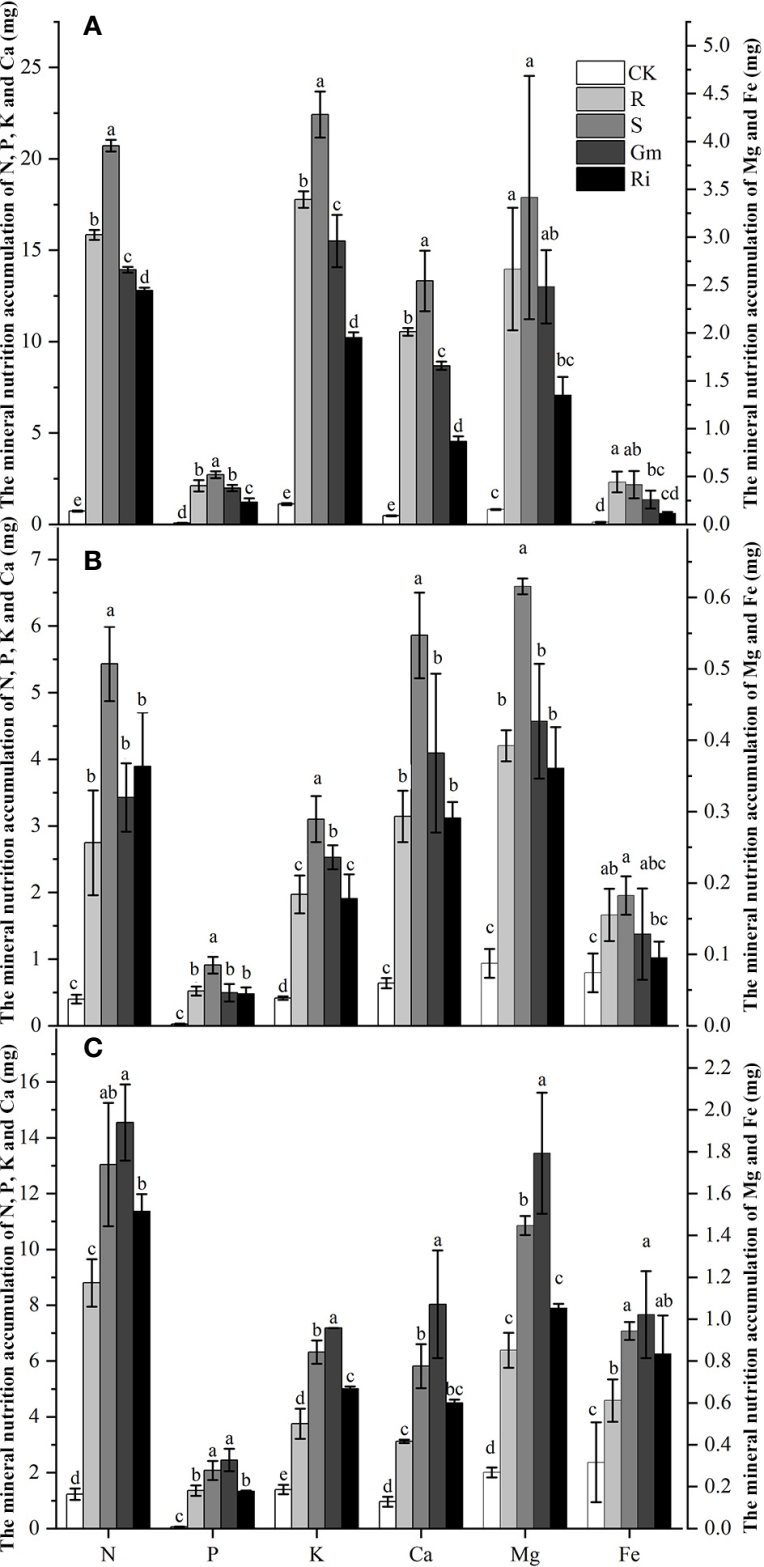
The mineral nutrition accumulation of *Pyrus betulaefolia* in different treatment. **(A)** Mineral nutrition accumulation of leaves **(A)**, stem **(B)** and root **(C)**. CK, sterilized soil from CK in *Trifolium repens* mixed with sterilized standard soil; R, soil from R in *Trifolium repens* mixed with sterilized standard soil; S, soil from S in *Trifolium repens* mixed with sterilized standard soil; Fm, *Funneliformis mosseae* inoculum mixed with sterilized standard soil; Ri, *Rhizophagus intraradices* inoculum mixed with sterilized standard soil. Data (means ± SD, n = 3) followed by different letters indicate that values are significantly different (*p* < 0.05) within the treatments.

The effects of diverse AMF taxa on mineral nutrients in leaves, stems and roots of pear plants were determined. AMF inoculation treatments resulted in higher mineral nutrient accumulation than that in CK treatment in all tissues (**Figure 2**). Indigenous AMF treatment promoted mineral accumulation to a greater extent than the commercial AMF isolates in shoot, but the commercial AMF isolates resulted in better mineral accumulation in the root (**Figure 2**).

### The metabolic responses in pear roots to diverse AMF taxa

In this study, 410 metabolites were detected in pear roots: mainly flavonoids, organo-oxygen compounds, prenol lipids, carboxylic acids and derivatives, glycerophospholipids, benzene and substituted derivatives, isoflavonoids, benzopyrans, coumarins and derivatives, organo-nitrogen compounds, steroids and steroid derivatives, glycerolipids and 2-arylbenzofuran flavonoids (**Figure S4**). In order to investigate differences in root metabolism, we focused on flavones, isoflavones, fatty acids, carbohydrates, amino acids, glycerolipids and glycerophospholipids that significantly differed across treatments.

**Figure 3:**
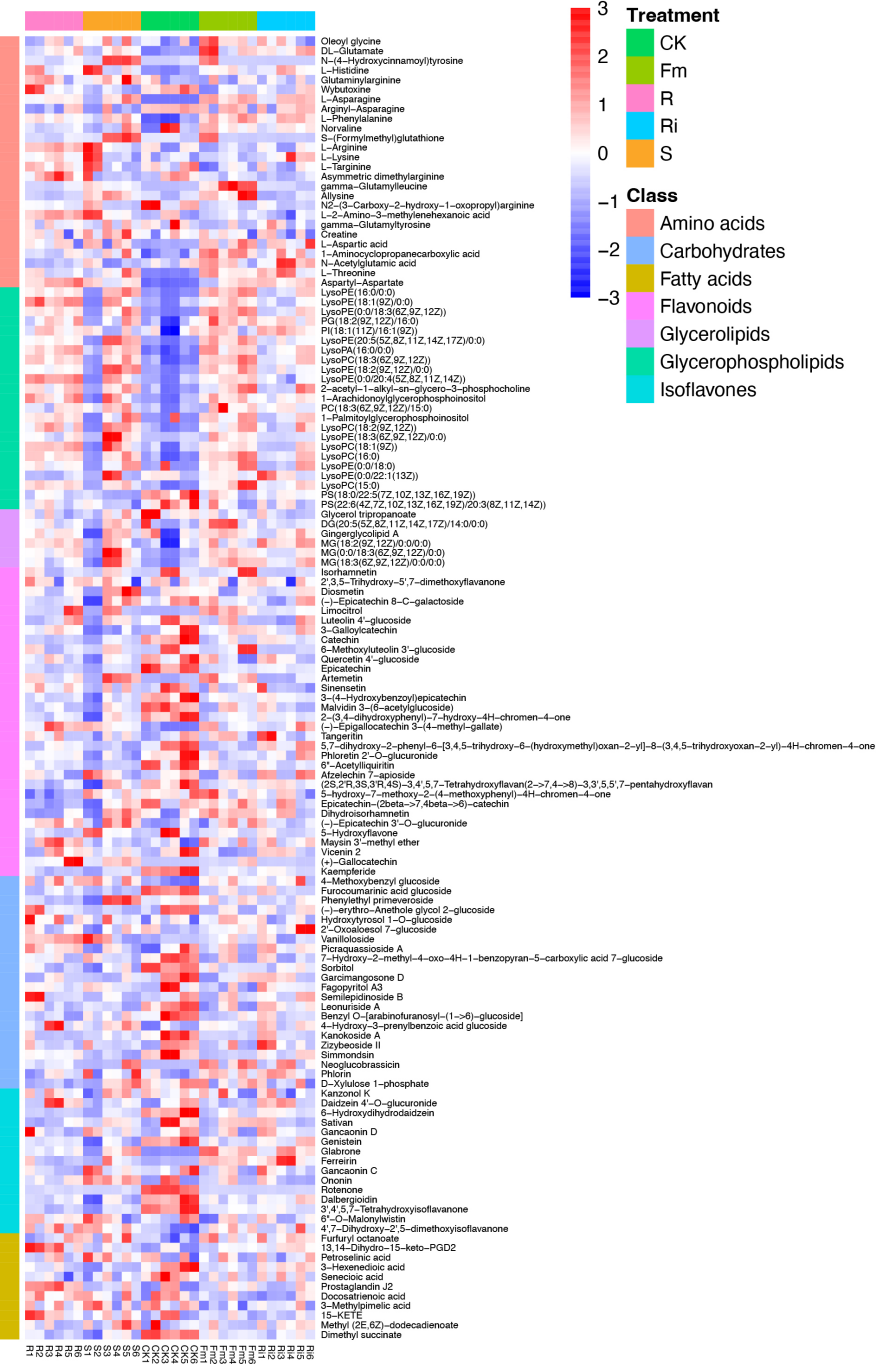
Heatmap of metabolites in *Pyrus betulaefolia* root. CK, sterilized soil from CK in *Trifolium repens* mixed with sterilized standard soil; R, soil from R in *Trifolium repens* mixed with sterilized standard soil; S, soil from S in *Trifolium repens* mixed with sterilized standard soil; Fm, *Funneliformis mosseae* inoculum mixed with sterilized standard soil; Ri, *Rhizophagus intraradices* inoculum mixed with sterilized standard soil.

In this study, compared with CK, multiple types of flavones, isoflavones, and carbohydrates decreased, whereas fatty acids, amino acids, glycerolipids, and glycerophospholipids increased in the AMF treatments (**Figure 3**).

Interestingly, the relative abundance of amino acids, glycerolipids, flavonoids, isoflavones, and fatty acids did not exhibit consistent changes in response to treatments. (**Figure 3** and **Table 4**). Compared with CK, the relative abundance of flavonoids, carbohydrates, and fatty acids were significantly decreased in R, S, *Fm* and *Ri* treatments (**Table 4**). In addition, the relative abundance of amino acids, glycerophospholipids and glycerolipids in the AMF treatment were higher than CK (**Table 4**).

**Table 4:** The relative abundance of metabolite on root of *Pyrus betulaefolia*.

Treatment	amino acids (%)	glycerophospholipids (%)	glycerolipids (%)	flavonoids (%)	carbohydrates (%)	isoflavones (%)	fatty acids (%)
CK	48.77 ± 9.11b	0.38 ± 0.14b	0.0188 ± 0.0057b	4.92 ± 1.23a	5.89 ± 0.86a	0.65 ± 0.13bc	1.14 ± 0.19a
R	65.36 ± 1.75a	0.82 ± 0.06a	0.0282 ± 0.0027a	1.25 ± 0.21c	2.86 ± 0.47c	0.52 ± 0.04c	0.58 ± 0.07bc
S	65.88 ± 13.53a	0.80 ± 0.44a	0.0341 ± 0.0219a	1.35 ± 0.75c	2.87 ± 0.90c	0.61 ± 0.36bc	0.57 ± 0.16c
Fm	55.05 ± 3.37b	1.04 ± 0.16a	0.0401 ± 0.0032a	1.83 ± 0.51bc	3.79 ± 0.45b	0.99 ± 0.21a	0.74 ± 0.06b
Ri	56.79 ± 5.04ab	0.77 ± 0.13a	0.0329 ± 0.0026ab	2.39 ± 0.64b	3.72 ± 0.60b	0.86 ± 0.28ab	0.73 ± 0.11bc

CK, sterilized soil from CK in *Trifolium repens* mixed with sterilized standard soil; R, soil from R in *Trifolium repens* mixed with sterilized standard soil; S, soil from S in *Trifolium repens* mixed with sterilized standard soil; Fm, *Funneliformis mosseae* inoculum mixed with sterilized standard soil; Ri, *Rhizophagus intraradices* inoculum mixed with sterilized standard soil. Data (means ± SD, n = 6) followed by different letters indicate that values are significantly different (*p* < 0.05) within the column.

The relative abundance of amino acids, flavonoids and carbohydrates showed different patterns between indigenous AMF and commercial AMF isolate treatments. The relative abundance of amino acids was higher in R and S, compared to *Fm* and CK treatments. The flavonoids and carbohydrates decreased significantly in the order, indigenous AMF, followed by specific isolates, and then by CK treatment.

### Differential transcriptome responses in pear root between indigenous AMF and commercial AMF isolates

To further compare the differences at the transcriptional levels between indigenous AMF and commercial AMF isolates, we sequenced the transcriptome of *Fm* and S, which resulted in better growth than other treatments, but also displayed significant differences in their metabolomes.

In the transcriptome 29,735,723 and 29,576,461 clean reads were obtained from the S and *Fm* root sample and accounted for 88.64% and 88.59% of the total sequences, respectively (**Table S1**). The reference genome for transcriptome analysis was the Chinese white pear (*P. bretschneideri*), and it resulted in matches of 76.30% and 76.56% of gene sequences for S and *Fm*, respectively (**Table S1**). We found a total of 1,217 differentially expressed genes (DEGs) for *Fm*/S, which included 682 up-regulated genes and 535 down-regulated genes (**Figure S5**).

**Figure 4:**
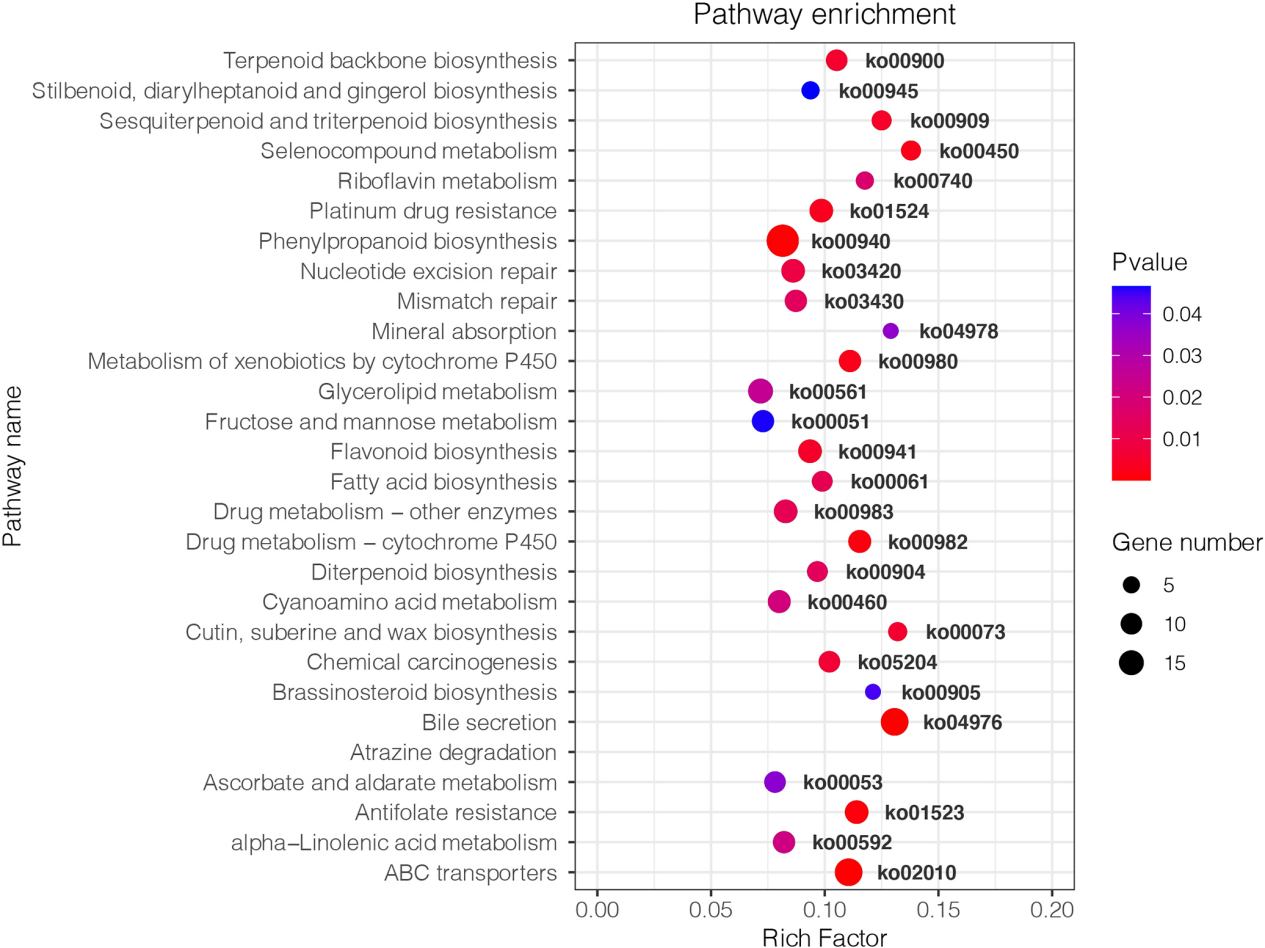
Kyoto Encyclopedia of Genes and Genomes (KEGG) enrichment analysis of DEGs in *Pyrus betulaefolia* roots between Fm and S. The size of the points represents the number of differentially expressed genes, and different colors represent different *P*-values. The larger the Rich Factor is the higher the degree of enrichment. S, soil from S in *Trifolium repens* mixed with sterilized standard soil; Fm, *Funneliformis mosseae* inoculum mixed with sterilized standard soil.

KEGG analysis showed that the DEGs between *Fm* and S were primarily involved in metabolic pathways (**Figure 4**). The transcripts associated with metabolic pathways of fructose and mannose metabolism (ko00051), fatty acid biosynthesis (ko00061) and flavonoid biosynthesis (ko00941), were significantly different between *Fm* and S treatments. We found 7 DEGs in *Fm* compared with S related to the fructose and mannose metabolism; these genes encoded enzymes such as mannose-6-phosphate isomerase (MPI), diphosphate-dependent phosphofructokinase (PFP), fructose-bisphosphate aldolase, class I (ALDO), triosephosphate isomerase (TPI) and mannan endo-1,4-beta-mannosidase (MAN) (**Table 5** and **Figure S6**). Compared to the S treatment, *MAN* (LOC103948539, LOC103960198), *MPI* (LOC103937460, LOC103948500), *ALDO* (LOC103934930) and *TPI* (LOC103965560) were significantly up-regulated by *Fm* treatment, while the *PFP* (LOC103955841) was significantly down-regulated. In the fatty acid biosynthesis pathway, the gene for the key enzyme of fatty acid biosynthesis, 3-oxoacyl-[acyl-carrier protein] reductase (*FabG*, LOC103960485), was significantly up-regulated and the acyl-[acyl-carrier-protein] desaturase (*Fab2*, LOC103939571) and long-chain acyl-CoA synthetase (*FadD*, LOC103964146) was down-regulated in *Fm* treatment. Four genes encoding fatty acyl-ACP thioesterase B (*FATB*) showed a different expression patterns: up-regulation of LOC103941054 and LOC103947075 and down-regulation of LOC103953493 and LOC103950938 (**Table 5** and **Figure S7**). In the flavonoid biosynthesis pathway, compared to S, the genes encoding 5-O-(4-coumaroyl)-D-quinate 3’-monooxygenase (*C3’H*, LOC103944997), shikimate O-hydroxycinnamoyltransferase (*HCT*, LOC103960625), caffeoyl-CoA O-methyltransferase (CCoAOMT, LOC103965012) and flavonol synthase (*FLS*, LOC103930998 and LOC103961089) were up-regulated in *Fm* and the HCT(LOC103940185), bifunctional dihydroflavonol 4-reductase (*DFR*, LOC103954960) and *FLS* (LOC103938099) were down-regulated (**Table 5** and **Figure S8**).

**Table 5:** KEGG enrichment analysis of fructose and mannose metabolism, fatty acid biosynthesis and flavonoid biosynthesis pathway in *Pyrus betulaefolia* roots between Fm and S.

KEGG ID	Pathway	geneID	ko_ID	ENZYME	Name	Symbol	FPKM_Fm	FPKM_S	log2FC
ko00051	Fructose and mannose metabolism	LOC103948539	K19355	3.2.1.78	mannan endo-1,4-beta-mannosidase	MAN	10.84	0	11.28
		LOC103960198					11.94	0.4975	4.65
		LOC103965560	K01803	5.3.1.1	triosephosphate isomerase	TPI	36.79	5.965	2.68
		LOC103937460	K01809	5.3.1.8	mannose-6-phosphate isomerase	MPI	26.65	9.38	1.56
		LOC103948500					17.92	8.6125	1.11
		LOC103934930	K01623	4.1.2.13	fructose-bisphosphate aldolase, class I	ALDO	24.25	12.085	1.09
		LOC103955841	K00895	2.7.1.90	diphosphate-dependent phosphofructokinase	PFP	0.4	12.335	-4.86
ko00061	Fatty acid biosynthesis	LOC103941054	K10781	3.1.2.21	medium-chain acyl-[acyl-carrier-protein] hydrolase	FATB	37.5375	7.905	2.30
		LOC103947075					26.865	9.7475	1.51
		LOC103953493					65.245	143.09	-1.06
		LOC103950938					65.245	143.09	-1.06
		LOC103960485	K00059	1.1.1.100	3-oxoacyl-[acyl-carrier protein] reductase	FabG	14.675	6.61	1.21
		LOC103964146	K01897	6.2.1.3	long-chain acyl-CoA synthetase	FadD	13.575	33.13	-1.23
		LOC103939571	K03921	1.14.19.2	acyl-[acyl-carrier-protein] desaturase	FAB2	7.0875	23.48	-1.66
ko00941	Flavonoid biosynthesis	LOC103965012	K00588	2.1.1.104	caffeoyl-CoA O-methyltransferase	CCoAOMT	62.32	0.2575	7.92
		LOC103944997	K09754	1.14.14.96	5-O-(4-coumaroyl)-D-quinate 3’-monooxygenase	C3’H	46.1575	17.08	1.50
		LOC103930998	K05278	1.14.20.6	flavonol synthase	FLS	26.72	2.31	3.57
		LOC103961089					21.4075	9.835	1.17
		LOC103938099					10.265	26.69	-1.21
		LOC103960625	K13065	2.3.1.133	shikimate O-hydroxycinnamoyltransferase	HCT	17.055	8.325	1.09
		LOC103940185					21.7875	57.09	-1.33
		LOC103954960	K13082	1.1.1.219	bifunctional dihydroflavonol 4-reductase/flavanone 4-reductase	DFR	9.895	57.33	-2.47

S, soil from S in *Trifolium repens* mixed with sterilized standard soil; Fm, *Funneliformis mosseae* inoculum mixed with sterilized standard soil (n=4, FPKM ≥ 10; Fragments per kilobase per million reads; *p* < 0.05).

## Discussion

### Propagation of indigenous AMF throuth different ways

In this study, we found that the mycorrhizal colonization in white clover root was significantly higher in S treatment, compared with the R treatment, and the accumulation of N, P, and K and biomass showed no differences, suggesting that the microorganisms in the root from pear plant could not assist the AMF proliferation. White clover may reshape the rhizosphere microorganism taxa through its root exudates to enrich its preferred microorganisms, resulting in microbial composition differences between propagation soil and original soil sample (Blažková et al., 2021). However, such changes may be transient, as the microorganism species ratios converged towards the spontaneously established composition within one plant generation (Blažková et al., 2021). In addition, the results also revealed there were no significant differences among all treatments for the diversity of bacterial communities (**Figures S2**, **S3**), indicating that bacterial communities tend to be identical through plant root selection and environmental factors in the process of culture (Fitzpatrick et al., 2018).

### Effects of indigenous and commercial AMF isolates on plant growth

AMF form symbiotic associations with plant roots and enhance nutrient uptake, plant growth and tolerance to biotic and abiotic stresses (Berger and Gutjahr, 2021). The ability of plants to react to AMF with changes in morphology and/or performance in terms of yield is termed ‘AM responsiveness’ (Berger and Gutjahr, 2021). The effects of commercial AMF isolates on plants are often affected by a variety of factors (such as organic matter concentration, pH, total N, available P concentration, texture of soil, climate and climate) (Pellegrino et al., 2015; Islam et al., 2021). Previous studies have shown that commercial AMF does not always show growth-promoting effects, and sometimes requires long time for colonization due to various factors (Li et al., 2019; Islam et al., 2021). However, the commercial AMF can strongly affect the local AMF communities and change their original function; some commercial AMF may even inhibit the diversity of AMF community (depending on phylogenetic relatedness and life history strategies) (Mummey et al., 2009). In this study, plant performance in terms of growth parameters and mineral nutrition accumulation in the AMF treatment were greater than under no-AMF treatment, wherein indigenous AMF performed better than single isolates. Meanwhile, the effects on pear plant growth, root architecture and colonization of *Fm* and S were better than other treatments (*Ri* and R treatments), which can be attributed to compatibility. Such compatibility between AMF and plant has been previously noted among commercial AMF isolates belonging to different species, as well as among isolates of the same species (Jansa et al., 2008). The AM responsiveness of plants varied from negative to positive depending on plant and AMF species, mainly because of the mutual selection of the plant and the AMF owing to compatibility. Thus, in this study similar selection may allow for the pear plant to establish efficient symbionts with *Fm* rather than *Ri*.

A mixture of AMF species that are complementary in their functions provide greater benefit for the plant than any single species. High diversity of indigenous AMF provide more benefits for nutrient uptake, drought tolerance, and pathogen protection rather than commercial AMF isolates (Wagg et al., 2011; Berruti et al., 2015; Johnson and Gibson, 2020), because it allows for plants to select appropriate cooperative partners to minimize carbon consumption and maximize acquisition of soil resources (Whiteside et al., 2019). The benefits on pear growth of S treatment were higher than that for R in the present study, which is consistent with our first hypothesis. A possible explanation may be that the microorganisms enriched in the root of pear may also inhibit the formation of mycorrhiza. A previous study suggests that abiotic and biotic factors in soil can inhibit mycorrhizal activities (Cruz-Paredes et al., 2020). There was no difference in the alpha- and beta-diversity of bacterial communities among treatments (**Figures S2**, **S3**), indicating differences in bacterial communities may not be a primary reason for higher mycorrhizal colonization in both clover and pear trees in soil without roots. In addition, active compounds, such as soluble phenolic compounds from plant litter can also affect the colonization of AMF due to their toxicity effects. However there are only a few studies on soluble phenolic compounds from litter and their inhibitory effects on AMF abundance (Piotrowski et al., 2008). Such active compounds may stimulate antagonistic fungi that compete for space and nutrients, or result in a combination of both where AMF are suppressed and other fungi capable of phenolic detoxification proliferate (Piotrowski et al., 2008). In addition, niche competition between AMF and other fungi also affects AMF colonization. Such interactions warrant further studies (Xie et al., 2021). In general, indigenous AMF are more adapted to the local environment and thus show prominent effects in terms of growth improvement. However, in some ways, *Fm* appeared to work better, which may be related to differences in microbes in natural soils, which may inhibit the growth of pear trees (LÜ et al., 2018).

Interestingly, we found that the root mineral nutrient accumulation in *Fm* was higher than in S, but the shoot mineral nutrition accumulation, including leaves and stem, was lower. The discrepancies in the effects of mineral nutrients accumulation between the shoot and root may be attributed to the effects of the AMF community which directly regulate root metabolism or due to differences in root to shoot partitioning. This phenomenon indicates that some AMF taxa in indigenous AMF could promote translocation of nutrients in plants. A previous study reported that AMF could enhance the isotopic fractionation of *Acacia caven* xylem water, indicating that AMF can not only affect the plant xylem transport, but also result in selective transport of mineral nutrients (Poca et al., 2019). These findings support the second hypothesis that high AMF diversity is more effective than commercial AMF isolates in pear plant as a suite of complementary functions owing to greater diversity may allow for more comprehensive promotion of pear tree growth.

### Metabolic responses in pear root between indigenous AMF and commercial AMF isolates

To further investigate the effects of indigenous AMF and commercial AMF isolates on plant root metabolism, we analyzed plant root metabolites across all treatments. In this study, AMF-inoculation mainly affected the accumulation of particular types of flavones, isoflavones, fatty acids, carbohydrates, amino acids, glycerolipids and glycerophospholipids. Results showed that AMF could decrease the flavonoids and carbohydrates and increase amino acids, glycerophospholipids and glycerolipids including their relative abundance. *R. irregularis* is a fatty acid auxotroph and fatty acids synthesized in the host plants are transferred to the fungus to sustain mycorrhizal colonization (Jiang et al., 2017). AMF uses lipids in roots as carbon sources, as well as sugars, which may promote the transformation of carbohydrates in roots into fatty acids. Subsequently, the lipid export pathway contributes a substantial amount of carbon to the AMF. This explains the reduced metabolite type and relative abundance of carbohydrates in the AMF treatment. Meanwhile, due to the consumption of fatty acids by AMF, the relative abundance of total fatty acids in roots is likely reduced.

The accumulation of certain flavonoids in mycorrhizal plants not only displayed time-specificity, but were also controlled by AMF species (Larose et al., 2002). Tian et al. (2021) found that higher concentrations of flavonoids (quercetin) in root exudates could enhance AMF colonization and biomass, indicating quercetin might be a key chemical signal affecting AM fungal associations (Tian et al., 2021). In our study, quercetin 4’-glucoside was higher in CK treatment than other AMF treatments (**Figure 3**), which could be attributed to the roots secreting specific flavonoids into the soil to induce mycorrhizal colonization, and reshaping of the AMF community. Moreover, flavonoids also have many other functions, including UV protection, as antioxidants, pigments, auxin transport regulation, defense against pathogens and signaling (Hassan and Mathesius, 2012). The flavonoids secretion of root enhanced by AMF is conducive to improve the adaptation of plants to the environment, and plays an important role in stabilizing the rhizosphere microorganisms and maintaining dynamic equilibrium.

The relative abundance of amino acids and carbohydrates showed substantially different trends between indigenous AMF treatment and commercial AMF isolates treatment. The higher relative abundance of amino acids in R and S and lower carbohydrates suggested that indigenous AMF enhanced the process of converting carbohydrates into amino acids, indicating indigenous AMF is more conducive to the accumulation of nitrogenous compounds and growth of the root system. Meanwhile, the relative abundance of flavonoids was higher in single isolates treatment, suggesting that single isolates required greater flavonoid biosynthesis in pear roots to enhance colonization than did the indigenous AMF. These results indicate that greater diversity in communities of indigenous AMF allow for functional complementarity within communities (Maherali and Klironomos, 2007).

### Differential transcriptome responses in pear root between indigenous AMF and commercial AMF isolates

Although indigenous AMF and commercial AMF isolates played a similar role in promoting pear tree growth, there were significant differences in metabolic pathways affected by them (**Figure 3** and **Table 4**). The diversity of metabolome between indigenous AMF treatment and commercial AMF isolates treatment may be associated with differences in the root transcriptomes. To test our third hypothesis, we focused on the DEGs in the pear root in response to *Fm* and S treatments. The DEGs were enriched in fructose and mannose metabolism, fatty acid biosynthesis, and flavonoid biosynthesis pathways. The gene expression of *MAN* was up-regulated by around 11-and 5-fold and *PFP* was down-regulated by around 5-fold in the *Fm* compared to S suggesting that the encoded enzymes respond to different types of AMF. As a key modifying enzyme of mannans, MAN plays an important role in cell wall hydrolysis, endosperm weakening in seedling development, endosperm degradation after the completion of seed germination and anther and pollen development (Yuan et al., 2007). *MAN* up-regulation may enhance cell wall hydrolysis and contribute to modifying root morphology in the *Fm* treatment (Inukai et al., 2012). PFP catalyzes reversible interconversion of Fructose 6-phosphate to Fructose 1,6-bisphosphate and may function in glycolysis to facilitate the survival of plants in low Pi environments by recycling Pi from PPi and conserving limited ATP pools in roots of plants that do not form symbiotic associations with mycorrhizal fungi (Murley et al., 1998). The P accumulation of root was higher in *Fm* compared to S suggesting *Fm* has an advantage over S in Pi absorption (**Figure 2**). Potentially, increase Pi availability which may down-regulate *PFP* expression.

**Figure 5:**
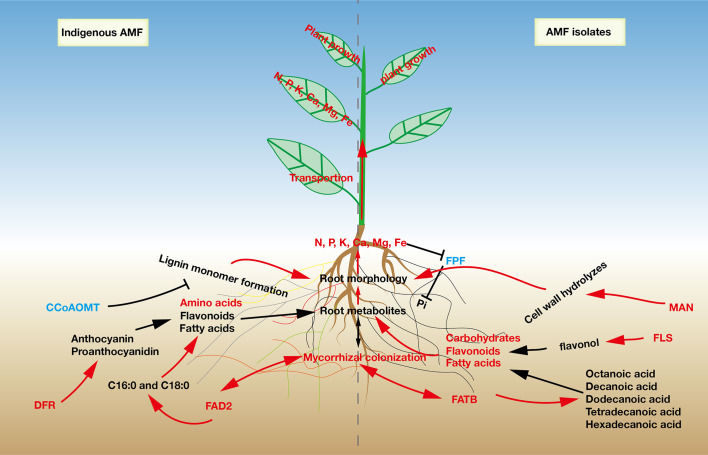
A proposed model to describe the effects of indigenous AMF and commercial AMF isolates on root responses in *Pyrus betulaefolia*.

Several key genes associated with fatty acid biosynthesis were identified as DEGs in this study, including 3.1.2.14 (fatty acyl-ACP thioesterase B, FATB), 1.14.19.2 (acyl-[acyl-carrier-protein] desaturase, FAB2), 6.2.1.3 (long-chain acyl-CoA synthetase, FadD) and 1.1.1.100 (3-oxoacyl-[acyl-carrier protein] reductase, FabG). FAD2 in the endoplasmic reticulum catalyzes the desaturation of fatty acids attached to phospatidylcholine (PC) from PC-18: 1 to PC-18:2 (Okuley et al., 1994). It’s down-regulated expression likely resulted in inhibition of accumulation of hexadecenoic acid and octadecenoic acid in the *Fm*, compared to S treatment (**Table 5** and **Figure S5**). Meanwhile, expression of the gene coding for FATB was up-regulated, likely enhancing the accumulation of octanoic acid, decanoic acid, dodecanoic acid, tetradecanoic acid and hexadecanoic acid. Sugiura et al. (2020) used eight saturated or unsaturated fatty acids (C12 to C18) and two β-monoacylglycerols to determine whether AM fungi can grow on medium supplied with fatty acids or lipids under asymbiotic conditions, demonstrating that myristate boosts the asymbiotic growth of AMF and can also serve as a carbon and energy source (Sugiura et al., 2020). These results suggest that *Fm* treatment can inhibit the biosynthesis of C16:0 and C18:0 and enhance the synthesis of tetradecanoic acid (myristic acid) in pear roots compared with S (**Figure 5**). The accumulation of myristic acid could stimulate spore germination and build-up of AMF biomass (Rillig et al., 2020), suggesting similar responses in pear roots.

In the flavonoid biosynthesis pathway, we focused on the genes encoding *CCoAOMT*, *FLS* and *DFR*. *CCoAOMT* has been suggested to be involved in a parallel pathway to lignin monomer formation, which promotes secondary cell wall formation (Marita et al., 2003). Down-regulation of *CCoAOMT* inhibited the synthesis of the secondary cell wall and allowed for root elongation (Toorchi et al., 2009), indicating that S may regulate lignin monomer formation pathway thereby affecting root morphology. The synthesis of flavonol aglycones has long been attributed to FLS, while DFR drives flux away from flavonols into anthocyanin and proanthocyanidin biosynthesis (Owens et al., 2008). The gene encoding *FLS* was up-regulated and that for *DFR* was down-regulated in the *Fm* treatment, indicating that *Fm* induced flavonoid synthesis toward flavonol synthesis, while S treatment resulted in diversion of flavonoid synthesis toward anthocyanin and proanthocyanidin biosynthesis (**Figure 5**).

## Conclusion

To reduce the application of chemical fertilizers in pear production and to maintain the ecological health of the soil, we studied the effects of indigenous AMF and commercial AMF isolates on the differences in the growth of pear seedlings. In our study, we used two different AMF propagation methods (R and S) to simulate spore propagation in natural soils. After propagation, root mycorrhizal colonization and arbuscular abundance in S was significantly higher than in R, indicating that the microorganisms in the root from pear plant could limit AMF proliferation, which is consistent with our first hypothesis. These results showed that the inoculum with the plant root may contain plant-specific metabolites or indigenous microorganisms that inhibit AMF colonization of the next host plant. We also found that the combined effects of indigenous AMF on pear tree were better than that of commercial AMF isolates on plant growth, root morphology, and mineral nutrient accumulation. Analyses of transcriptomes and metabolomes demonstrated that pear roots displayed specific changes in metabolism and gene expression to respond to indigenous AMF and commercial AMF isolates, especially in the metabolism of flavones, carbohydrates and fatty acids. Taken together, our findings demonstrate the response of pear seedlings to indigenous AMF and commercial AMF isolates in aspects from the transcriptional level, metabolic capacity, mineral nutrient accumulation and plant growth, providing a new understanding of the relationship between AMF community and pear tree growth (**Figure 5**).

## Data availability statement

The datasets presented in this study can be found in online repositories. The names of the repository/repositories and accession number(s) can be found below: https://figshare.com/s/b81244b2c1bb72c49b2e and https://www.ncbi.nlm.nih.gov/, PRJNA761485.

## Author contributions

YX and CD conceived and designed the experiments; WF, HP and HL performed the experiments; YS, SJ, PL and RJ analyzed the data; YS and SJ wrote the manuscript; TC, SN, TY and GJ revised the manuscript. All authors contributed to the article and approved the submitted version.
