# Supplementary material for: Indigenous and commercial isolates of arbuscular mycorrhizal fungi display differential effects in Pyrus betulaefolia roots and elicit divergent transcriptomic and metabolomic responses
Source: Front Plant Sci. 2023 Jan 9;13:1040134. doi: 10.3389/fpls.2022.1040134 (PMC9868765; doi:10.3389/fpls.2022.1040134)
Supplement: Supplementary file 1 [file DataSheet_1.doc]

**Methods** **S1**

DNA was extracted using DNA extraction kit (MoBio Laboratories, Carlsbad, USA). The concentration and purity were measured using the NanoDrop One (Thermo Fisher Scientific, MA, USA). 16S rRNA genes of distinct regions (V4-V5) were amplified used specific primer (515F GTGCCAGCMGCCGCGGTAA，907R CCGTCAATTCMTTTRAGTTT ) with 12bp barcode. Primers were synthesized by Invitrogen (Invitrogen, Carlsbad, CA, USA). The length and concentration of the PCR product were detected by 1% agarose gel electrophoresis. PCR products with the correct expected length was mixed in equal identity ratios according to the GeneTools Analysis Software (Version4.03.05.0, SynGene). Then the mixture of PCR products was purified with E.Z.N.A. Gel Extraction Kit (Omega, USA).

Sequencing libraries were generated using NEBNext® Ultra^TM^ II DNA Library Prep Kit for Illumina® (New England Biolabs, MA, USA) following manufacturer's recommendations and index codes were added. The library quality was assessed on the Qubit@ 2.0 Fluorometer (Thermo Fisher Scientific, MA, USA). At last, the library was sequenced on an Illumina Nova6000 platform and 250 bp paired-end reads were generated (Guangdong Magigene Biotechnology Co.,Ltd. Guangzhou, China).

Sequences with ≥97% similarity were assigned to the same OTU (Operational Taxonomic Unit). For each representative sequence, the silva (https://www.arb-silva.de/), RDP ( http://rdp.cme.msu.edu/index.jsp), Greengenes (http://greengenes.lbl.gov/) database were used to annotate taxonomic information by usearch-sintax(set the confidence threshold to default to ≥ 0.8). The differences between groups were analyzed by alpha diversity index using R software, one-way ANOVA was used. OTU abundance was normalized before Beta diversity analysis. PCoA were displayed by vegan package in R software through bray_curtis method.


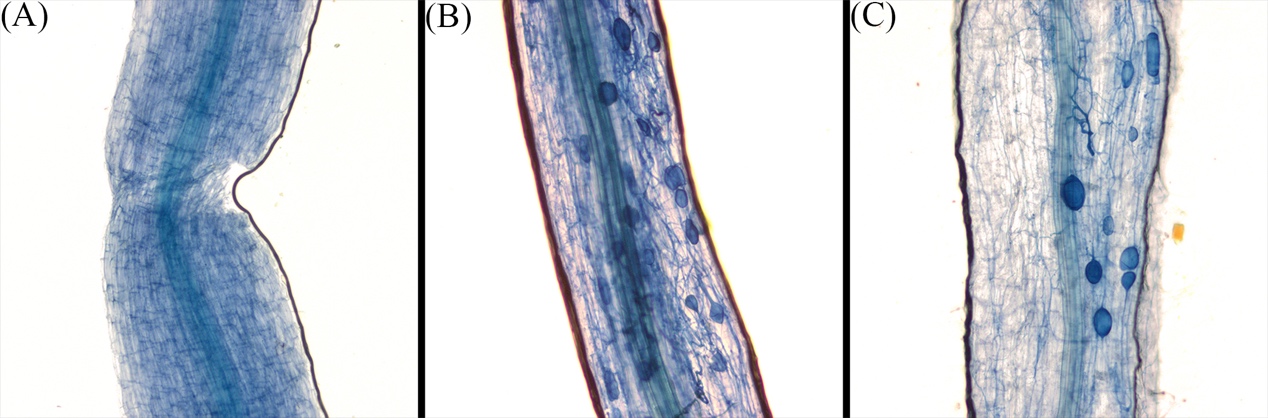


**FIGURE S1 | The root mycorrhizal colonization in the white clover root.** (A) the root mycorrhizal colonization in CK, (B) the root mycorrhizal colonization in R and (C) the root mycorrhizal colonization in S. CK, sterilized soil from rhizosphere mixed with sterilized sand and vermiculite; R, soil and fine roots from rhizosphere mixed with sterilized sand and vermiculite; S, only soil from rhizosphere mixed with sterilized sand and vermiculite.


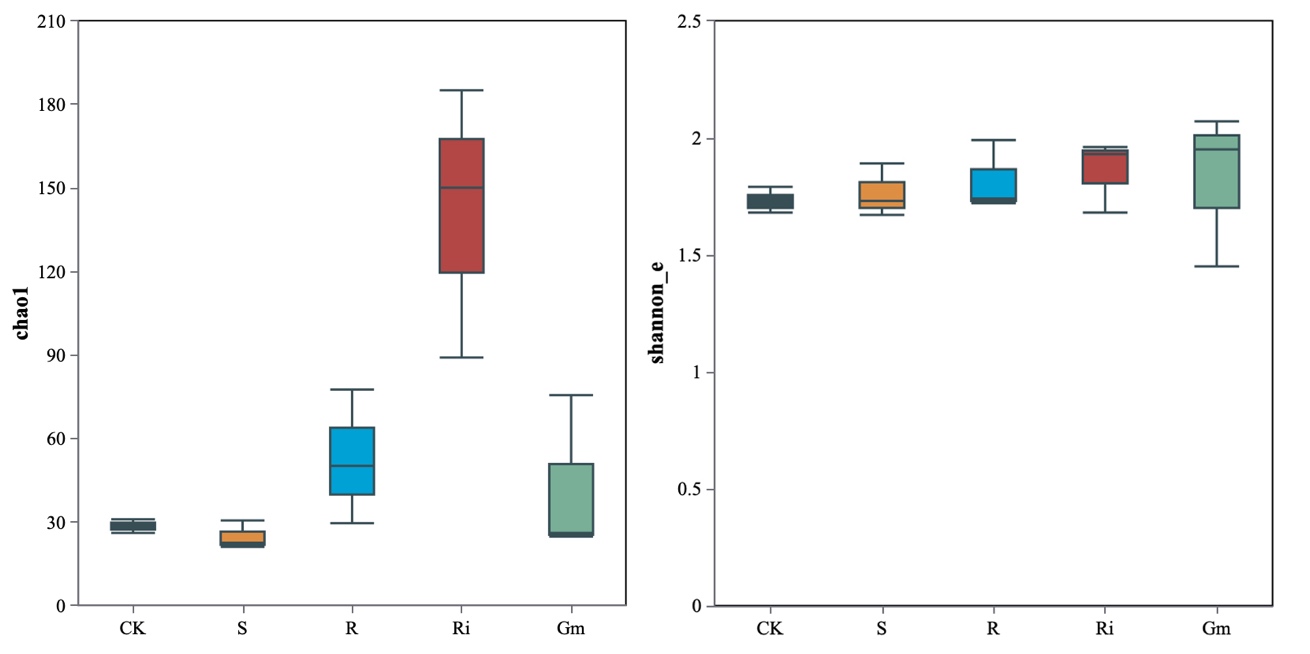


**FIGURE S2 | Bacterial community alpha-diversity (Chao1 and Shannon) in Genus level of rhizosphere soils among all treatment in *Pyrus betulifolia*.**


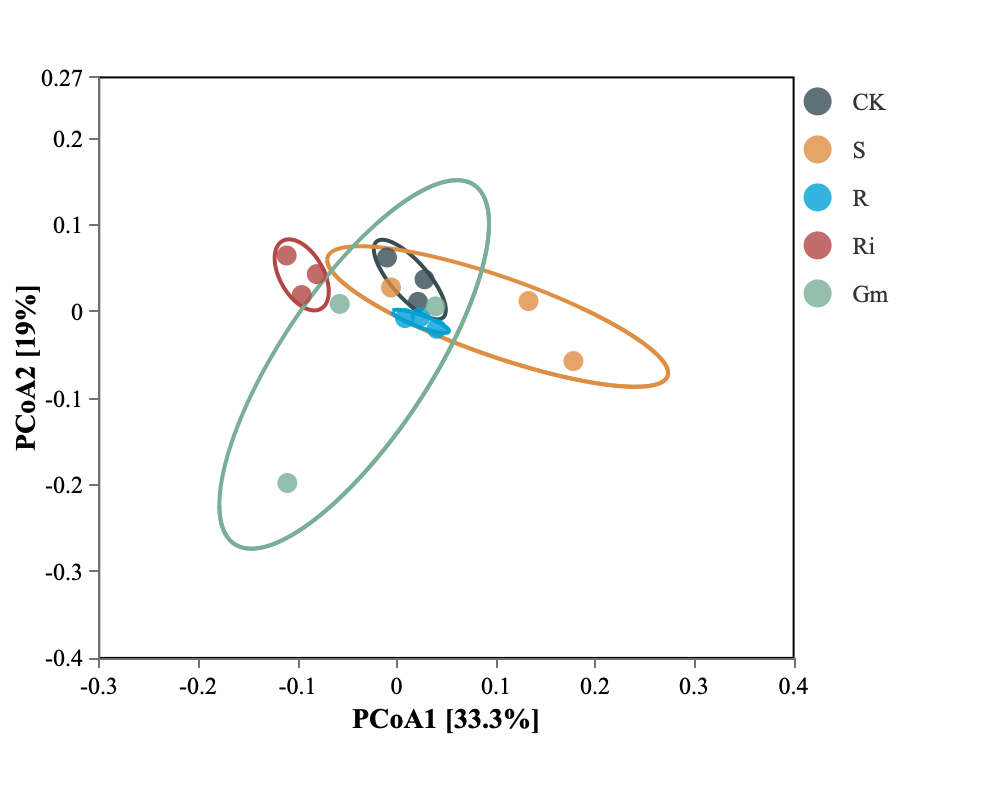


**FIGURE S3 | Bacterial community beta-diversity in Genus level of rhizosphere soils among all treatment in** ***Pyrus betulifolia*.**


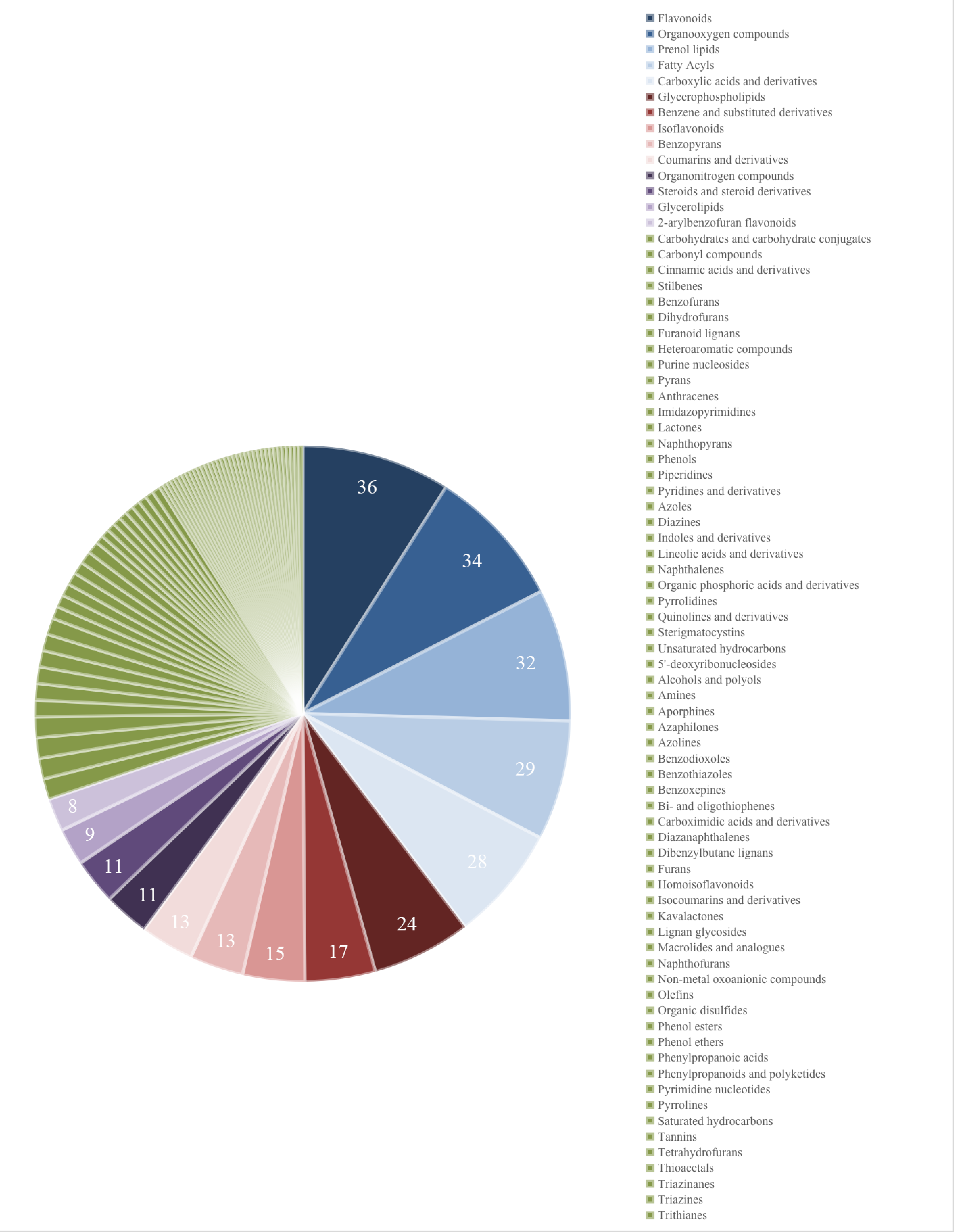


**FIGURE S4 | The metabolite profile of *Pyrus betulifolia* root.**


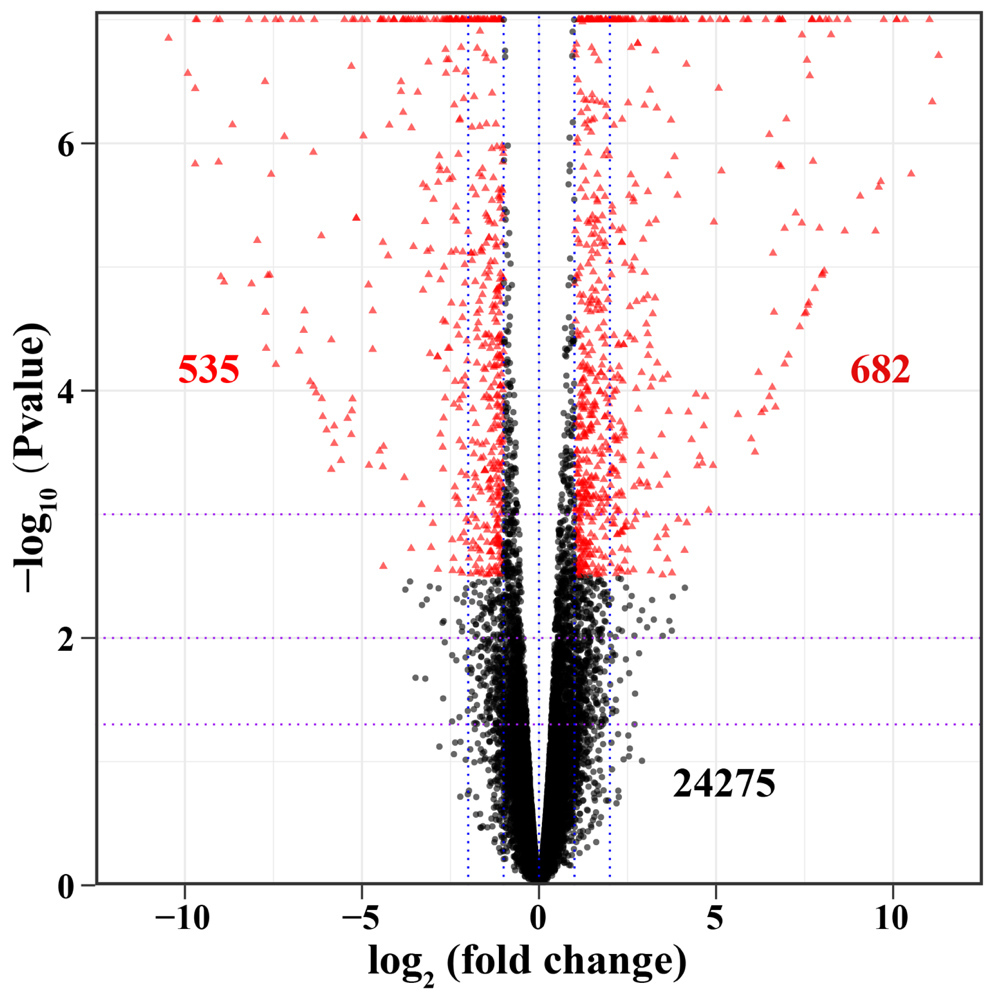
**FIGURE S****5 | Volcano plots of DEGs in transcriptome analysis of *Pyrus betulifolia* roots (FPKM ≥ 2; Fragments per kilobase per million reads).**

**FIGURE S****6 | KEGG enrichment analysis of fructose and mannose metabolism pathway in *Pyrus betulifolia* roots.** ﻿Red and blue indicate related genes that were up- or down-regulated based on the transcriptome analysis (**FPKM ≥ 10; Fragments per kilobase per million reads**).
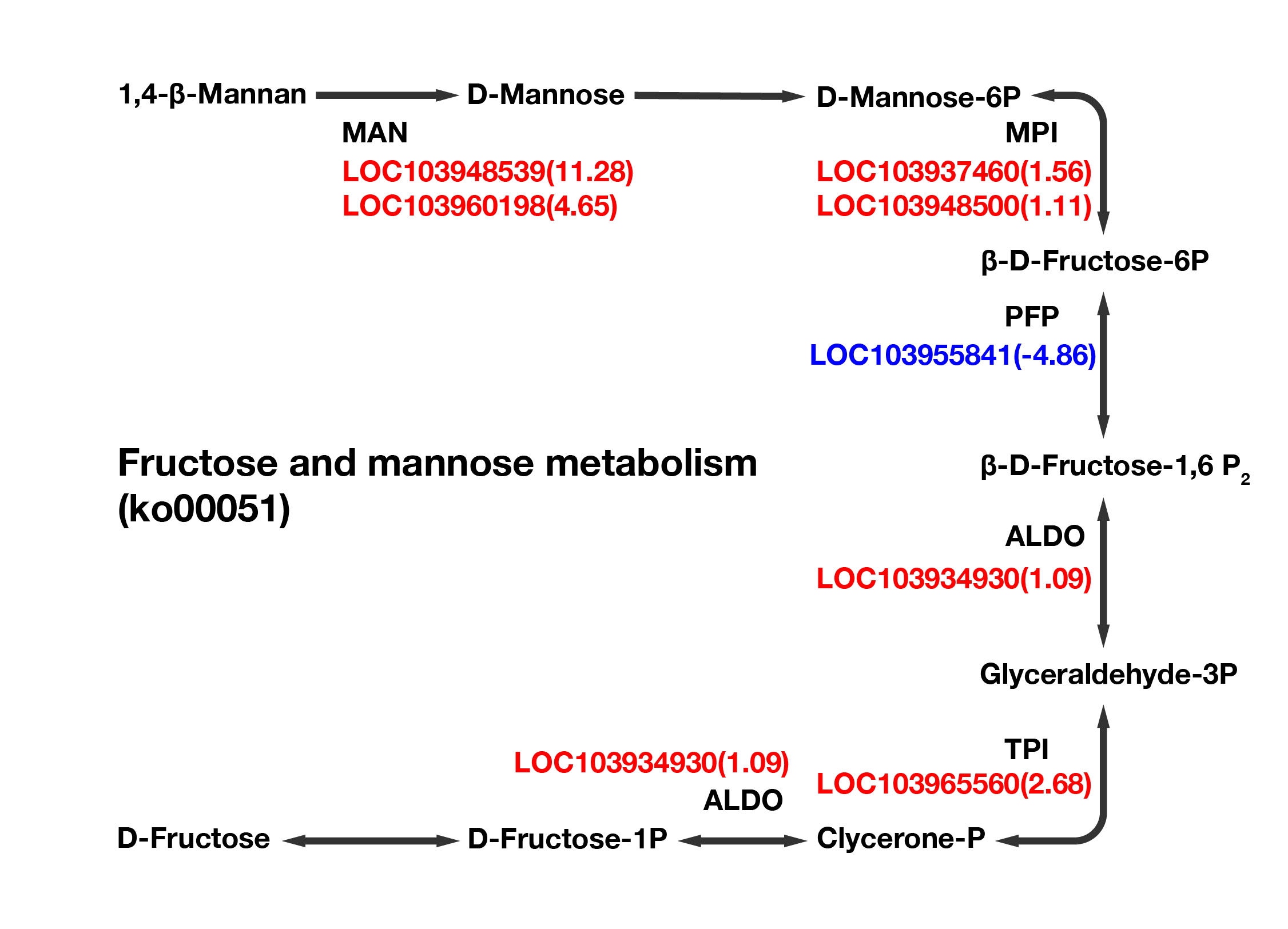


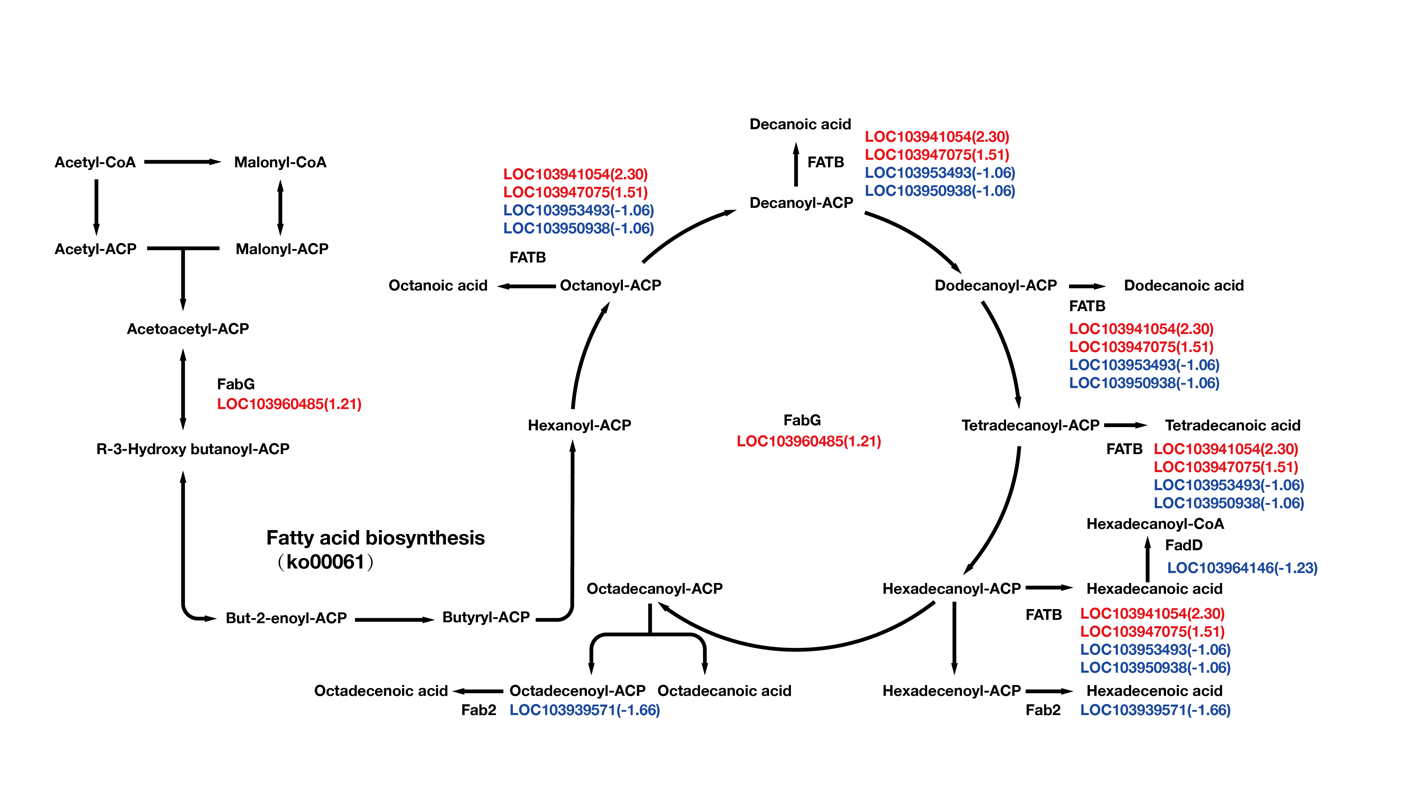
**FIGURE S7 | KEGG enrichment analysis of** **fatty acid biosynthesis pathway in *Pyrus betulifolia* roots.** Red and blue indicate related genes that were up- or down-regulated based on the transcriptome analysis (**FPKM ≥ 10; Fragments per kilobase per million reads**).


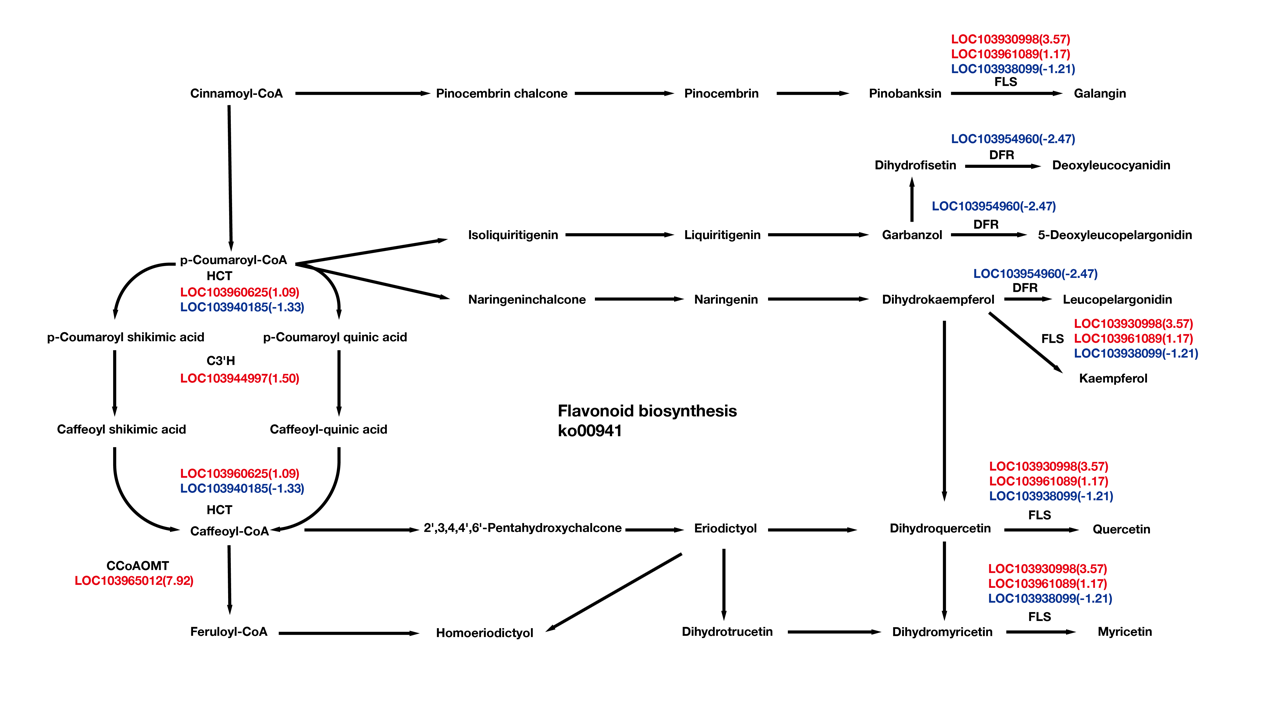
**FIGURE S8 | KEGG enrichment analysis of flavonoid biosynthesis pathway** **in *Pyrus betulifolia* roots.** Red and blue indicate related genes that were up- or down-regulated based on the transcriptome analysis (**FPKM ≥ 10; Fragments per kilobase per million reads**).

**TABLE S1 | Summary of RNA-seq quality information**

| Sample | Clean reads | Clean data ratio (%) | Reference genome mapped ratio (%) |
| --- | --- | --- | --- |
| S | 29735723 | 88.64 | 76.30 |
| Fm | 29576461 | 88.59 | 76.56 |

**TABLE S2 | Summary of statistical analysis information**

|  | df | Sum Sq | Mean Sq | F value | p value |
| --- | --- | --- | --- | --- | --- |
| Leaves number | 4 | 1303.5 | 325.9 | 15.07 | <0.001 |
| Plant height | 4 | 1287.7 | 321.9 | 45.34 | <0.001 |
| Shoot dry biomass | 4 | 1.4383 | 0.3596 | 9.07 | <0.001 |
| Root dry biomass | 4 | 0.8275 | 0.20687 | 10.49 | <0.001 |
| Leaf area | 4 | 447915258 | 1.12E+08 | 15.3 | <0.001 |
| Total length | 4 | 40.13 | 10.032 | 5.06 | <0.01 |
| Surface area | 4 | 10652 | 2662.9 | 9.113 | <0.001 |
| Root volume | 4 | 2.2317 | 0.5579 | 11.37 | <0.001 |
| Leaf N | 4 | 657.2 | 164.3 | 3648 | <0.001 |
| Leaf P | 4 | 12.393 | 3.0982 | 74.33 | <0.001 |
| Leaf K | 4 | 798.3 | 199.57 | 254 | <0.001 |
| Leaf Ca | 4 | 307.72 | 76.93 | 132 | <0.001 |
| Leaf Mg | 4 | 19.49 | 4.872 | 11.05 | <0.01 |
| Leaf Fe | 4 | 0.4039 | 0.10098 | 12.27 | <0.001 |
| Stem N | 4 | 40.69 | 10.172 | 27.38 | <0.001 |
| Stem P | 4 | 1.1825 | 0.29562 | 31.25 | <0.001 |
| Stem K | 4 | 12.05 | 3.0125 | 40.8 | <0.001 |
| Stem Ca | 4 | 42.87 | 10.718 | 26.2 | <0.001 |
| Stem Mg | 4 | 0.431 | 0.10774 | 50.28 | <0.001 |
| Stem Fe | 4 | 0.02301 | 0.005753 | 3.884 | <0.05 |
| Root N | 4 | 329.7 | 82.43 | 52.27 | <0.001 |
| Root P | 4 | 10.064 | 2.5161 | 39.56 | <0.001 |
| Root K | 4 | 62.07 | 15.518 | 154.1 | <0.001 |
| Root Ca | 4 | 85.85 | 21.463 | 24.42 | <0.001 |
| Root Mg | 4 | 4.061 | 1.0154 | 54.27 | <0.001 |
| Root Fe | 4 | 0.9766 | 0.24415 | 9.759 | <0.01 |
| amino acids | 4 | 0.1265 | 0.03164 | 5.173 | <0.01 |
| glycerophospholipids | 4 | 0.0001374 | 3.43E-05 | 6.655 | <0.001 |
| glycerolipids | 4 | 1.52E-07 | 3.80E-08 | 3.538 | <0.05 |
| flavonoids | 4 | 0.005443 | 0.0013608 | 24.46 | <0.001 |
| carbohydrates | 4 | 0.00368 | 0.0009201 | 19.86 | <0.001 |
| isoflavones | 4 | 8.93E-05 | 2.23E-05 | 4.088 | <0.05 |
| fatty acids | 4 | 1.28E-04 | 3.20E-05 | 18.93 | <0.001 |
